# Characterization of Embryonic Skin Transcriptome in *Anser cygnoides* at Three Feather Follicles Developmental Stages

**DOI:** 10.1534/g3.119.400875

**Published:** 2019-12-02

**Authors:** Chang Liu, Cornelius Tlotliso Sello, Yujian Sui, Jingtao Hu, Shaokang Chen, Petunia Msuthwana, Yuxuan Zhou, Sulleyman Kassim Wachiebine, Yue Sun, Jing Liu, Shengyi Li, Wei Yang, Yupu Song, Yunpeng Xu, Chanying Guo, Qihui Sui, Yongfeng Sun

**Affiliations:** * College of Animal Science and Technology, Jilin Agricultural University, Changchun 130118, Jilin, China,; † Beijing General Station of Animal Husbandry, Beijing 100107, China, and; ‡ Key Laboratory for Animal Production, Product Quality and Safety of Ministry of Education, Changchun 130118, Jilin, China

**Keywords:** *Anser cygnoides* feathers, dynamic morphogenesis, *de novo* transcriptome sequencing, expression profiles, functional annotation

## Abstract

In order to enrich the *Anser cygnoides* genome and identify the gene expression profiles of primary and secondary feather follicles development, *de novo* transcriptome assembly of skin tissues was established by analyzing three developmental stages at embryonic day 14, 18, and 28 (E14, E18, E28). Sequencing output generated 436,730,608 clean reads from nine libraries and *de novo* assembled into 56,301 unigenes. There were 2,298, 9,423 and 12,559 unigenes showing differential expression in three stages respectively. Furthermore, differentially expressed genes (DEGs) were functionally classified according to genes ontology (GO), Kyoto Encyclopedia of Genes and Genomes (KEGG), and series-cluster analysis. Relevant specific GO terms such as epithelium development, regulation of keratinocyte proliferation, morphogenesis of an epithelium were identified. In all, 15,144 DEGs were clustered into eight profiles with distinct expression patterns and 2,424 DEGs were assigned to 198 KEGG pathways. Skin development related pathways (mitogen-activated protein kinase signaling pathway, extra-cellular matrix -receptor interaction, Wingless-type signaling pathway) and genes (delta like canonical Notch ligand 1, fibroblast growth factor 2, Snail family transcriptional repressor 2, bone morphogenetic protein 6, polo like kinase 1) were identified, and eight DEGs were selected to verify the reliability of transcriptome results by real-time quantitative PCR. The findings of this study will provide the key insights into the complicated molecular mechanism and breeding techniques underlying the developmental characteristics of skin and feather follicles in *Anser cygnoides*.

Application of molecular genetics technologies offers potential ways to assist in breeding and selection of animals for a variety of traits and to increase the accuracy of predicting individual mature phenotype from early stages of development (Alemayehu and Getu 2015). Breeding programs that involves the identification of potential target genes and allied genotypes can make an imperative contribution to the improvement of economic traits in poultry (Niu *et al.* 2017).

Waterfowl consumption level has increased over the past years, resulting in an ever-increasing market demand for their products (Pingel and Germany 2011). Feathers are the functional skin derivatives in birds that serve numerous functions such as tactile sensation, physical protection, thermoregulation and several types of observable roles such as demonstration in courtship and flight (Kondo *et al.* 2018). Birds’ feathers are categorized based on their structural appearance and functions on different regions of the body, the classifications are mainly: down feathers, contour feathers and flight feathers (Yu *et al.* 2004). Geese downy feathers have high economic value as they are used in high quality winter clothing and bedding with low bulk density and provide good insulation (Kozák *et al.* 2010). During the embryonic skin development, feather follicles with primordial follicle buds consist of sheets of epithelial cells attached to a portion of mesenchymal cells via a basement membrane (Shyer *et al.* 2017). Feather follicles are complex epidermal structures essential for growth and development of feathers by the reciprocating signal transmission between epidermis and dermis during the embryonic period (Inamatsu *et al.* 2006). Transduction molecules, transcription factors, and other genetic factors through the interrelated but relatively independent ways influence dynamic morphogenesis of skin and feather follicles during the complex developmental process of feather formation (Jiang *et al.* 2011). Previous studies reported the formation, morphogenesis, differentiation, and maturation of the skin and feather follicles (Yu *et al.* 2002; Lin *et al.* 2006; Chen *et al.* 2017). Recently, transcriptomic analysis have been conducted to identify candidate genes (gene expression patterns) involved in differentiation, growth regulation, survival, and morphogenesis of different types of feathers (in adults) and embryonic integuments of ducks, chicken and goose (*Anser anser*) (Ng and Li 2018; Liu *et al.* 2018; Lowe *et al.* 2015). Furthermore, Sello *et al.* (2019) only reported a single time point during the embryonic skin development performance evaluation transcriptome between *Anser anser* and *Anser cygnoides*. However, there are limited studies on the regulatory transcriptome information at different developmental stages of embryonic skin feather follicles in *Anser cygnoides* species.

Strand-specific RNA-sequencing (RNA-Seq) is a sophisticated next-generation sequencing technique that provides insights to understanding complex and multidimensional processes of molecular biology such as transcriptome to analyze global differentially expressed genes (DEGs) in a specified period (Zhang *et al.* 2012). In the present study, *de novo* transcriptome sequencing was done to provide large-scale genomic resources and to identify differentially expressed genes during the development of embryonic skin feather follicles in *Anser cygnoides*. In addition, Hematoxylin-eosin routine staining (H&E) was used to perform histological analysis to observe the developmental changes of geese embryonic skin feather follicles. The findings of this study would provide insights into potential genes and signaling pathways which might be the focus in future studies in marker-assisted selection and further help to improve downy feathers yield and other functional researches in amniotes.

## Materials and Methods

### Ethics statement

All experimental procedures were conducted in accordance with guidelines developed by the Ministry of Science and Technology of China (2006) and approved by the Goose Industry Research and Development Center of Jilin Agricultural University (Approval number: GR(J)18-008. Date: April 13, 2018).

### Sample collection

A total of 230 fertilized geese eggs were obtained from a mixed flock of *Anser cygnoides* in Jilin Agricultural University (Changchun, Jilin, Northeast of China). The eggs were incubated in an incubator with forced draft fan at a suitable temperature and humidity (Xu *et al.* 2007). Three of the incubated eggs were randomly selected and observed daily for embryonic feather development. Random selection of the eggs was done daily from the embryonic day 14 (E14) to embryonic day 28 (E28).

Six skin samples from the dorsum of geese embryos sampled at embryonic day 14 (E14), the primordial period of primary feather follicles); embryonic day 18 (E18), the primordial period of secondary feather follicles); and embryonic day 28 (E28), the greater developmental period of secondary feather follicles); were used for experimental analysis. Among them, three samples at each stage were immediately stored in vials containing 10% neutral buffered formalin, the remaining nine samples from three stages to be used for RNA extraction were immediately frozen in liquid nitrogen and then stored at -80° refrigerator.

### Hematoxylin and eosin staining

Skin tissues were immersed in formalin and embedded in paraffin. De-paraffinization with xylene in 2 periods for 5 min for rehydration was performed. Subsequently, hematoxylin solution was used to stain the 5-μm longitudinal sections for 5 min, following the treatment with 1% hydrochloric acid-ethanol and then the slides were washed by tap water. Consequently, Eosin solution and a grades series of ethanol were used to wash out the excessive dye and for onwards dehydration. Subsequently, each slide was treated with xylene for 10 min and sealed with neutral resin. The morphological changes of feather follicles were observed under Nicon-300 light microscope (Nicon, Tokyo, Japan).

### Total RNA isolation and Illumina sequencing

Total RNA was isolated from nine tissues using the TRIzol Reagent (Invitrogen Life Technologies, Carlsbad, CA, USA) following the instructions of the manufacturer. DNase I (Ambion, USA) was used to ensure that DNA contamination was removed from RNA samples. The quantity and integrity of total RNA were confirmed by Nanodrop spectrophotometer 2000 (Thermo Scientific, Wilmington, DE, USA) and Agilent 2100 Bioanalyzer (Agilent Technologies, Santa Clara, CA, USA). After total RNA was isolated, we used Oligo (dT) beads to enrich eukaryotic mRNA, and Ribo-ZeroTM Magnetic Kit (Epicentre) was used to enrich prokaryotic mRNA by removing rRNA. Subsequently, fragmentation buffer was employed to fragment the enriched mRNA into short fragments which were then reverse transcribed to cDNA by random primers. After the synthesis of second-strand cDNA with DNA polymerase I, RNase H, dNTP and buffer, the cDNA fragments were purified using QiaQuick PCR extraction kit (Qiagen, Hilden, Germany), end repaired, poly(A) added, and ligated to paired-end adapters (Illumina). The size of ligation products was visualized by agarose gel electrophoresis, and sequenced using an Illumina HiSeqTM 4000 (Gene Denovo Biotechnology, Guangzhou, China).

### De novo transcriptome assembly and annotation

Raw reads obtained from Illumina sequencing containing adapters or low quality bases which would affect the following assembly and analysis were filtered to gain clean reads by removing adapters. This was done by removing reads containing more than 10% of unknown nucleotides (N), and removing low quality reads containing more than 50% of low quality (Q-value ≤ 10) bases. Clean reads of the nine libraries were *de novo* assembled by Trinity software with default parameters to get the sequences defined as unigenes (Grabherr *et al.* 2011).

Unigenes were annotated using BLAST (Basic Local Alignment Search Tool) searches against the NCBI Nr protein database (NCBI non-redundant sequence database, available online: http://www.ncbi.nlm.nih.gov), Swiss-Prot (available online: http://www.expasy.ch/sprot), KEGG (the Kyoto Encyclopedia of Genes and Genomes, available online: http://www.genome.jp/kegg), and KOG (Eukaryotic Orthologous Groups, available online: http://www.ncbi.nlm.ih.gov/COG) with e-value cut-off of 1e-5, and the sequence direction of unigenes was used to get the optimal result. In the case of the conflict among different databases, we followed the priority order of Nr, Swiss-Prot, KEGG and KOG. Besides, we used Blast2GO software to obtain Gene ontology (GO, available online: http://www.geneontology.org/) annotations of the unigenes and then we used WEGO software (available online: http://wego.genomics.org.cn/cgibin/wego/index.pl) to distribute gene functions by performing GO functional classification.

### Quantification and analysis of differentially expressed genes (DEGs)

The number of clean reads was mapped into the reference database through the SOAPaligner/soap2 software and normalized to the RPKM (reads per kilobase per million mapped reads) to quantify the gene expression levels. The RPKM method is able to eliminate the influence of different gene length and sequencing data amount, which could be directly used for comparing the difference of gene expression among samples. Genes with a |Fold Change| ≥2 and a false discovery rate (FDR) <0.05 in a comparison were identified as significant DEGs by using edgeR package (available online: http://www.r-project.org/). DEGs were then subjected to enrichment analysis of GO functions and KEGG pathways. To examine the expression pattern of DEGs, the expression data (from the E14 to E28 stage) were normalized to log2^(E14/E14)^, log2^(E18/E14)^, and log2^(E28/E14)^, and then clustered by Short Time-series Expression Miner software (STEM) (Ernst and Bar-Joseph 2006). Additionally, the clustered profiles with p-value ≤0.05 were considered as significant profiles.

### Real-time quantitative PCR (RT-qPCR) validation

In order to confirm the reliability of RNA-Seq results, we selected eight DEGs (ectodysplasin A2 receptor, *EDA2R*; ribosomal protein S9, *RPS9*; aldolase, fructose-bisphosphate A, *ALDOA*; filamin A, *FLNA*; clathrin light chain A, *CLTA*; polo like kinase 1, *PLK1*; vasodilator stimulated phosphoprotein, *VASP*; and EH domain containing 2, *EHD2*) related to feather development to do the RT-qPCR analysis, in which we used the same RNA samples as previously described for transcriptome sequencing. Superscript first-strand synthesis system (Invitrogen, Shanghai, China) was used to generate the first-strand cDNA from 1 μg total RNA and then the cDNA samples were diluted 10 times prior to subsequent experimentation. The RT-qPCR reactions were performed on Applied Biosystems 7500 Real-Time PCR System (Thermo Fisher Scientific Inc.,Waltham, MA, USA) using SYBR Green Realtime PCR Master Mix (TOYOBO, Osaka, Japan). The study utilized Primer 5 (Primer-E Ltd, Plymouth, UK) to design gene-specific primers and regarded *β-actin* as internal reference. All the primers are listed in Table S1. Briefly, each reaction was performed with a total volume of 20 μL containing 2 μL of cDNA, 0.8 μL each of forward and reverse primers, 10 μL of SYBR Green Real-time PCR Master Mix, and 6.4 μL of distilled water, with pre-denaturation at 95° for 3 min, followed by 40 cycles of amplification (95° for 15 s, 60° for 15 s and 72° for 45 s). The 2^-ΔΔCt^ method was used to calculate the relative expression of each gene.

### Data availability

Supplemental files are available at figshare. Table S1: Primers used in RT-qPCR, Table S2: E-value and top 10 species distributions, Table S3: Unigenes assigned to 25 function categories, Table S4: DEGs with stage-specific up expression in E14 *vs.* E18, E18 *vs.* E28, and E14 *vs.* E28, Table S5: DEGs with stage-specific down expression in E14 *vs.* E18, E18 *vs.* E28, and E14 *vs.* E28, Table S6: DEGs in E14 *vs.* E18 were assigned to three major GO categories, Table S7: DEGs in E14 *vs.* E28 were assigned to three major GO categories. Table S8: DEGs in E18 *vs.* E28 were assigned to three major GO categories, Table S9: Expression of DEGs between the different species by the values of RPKM and Count. The Illumina reads were deposited in the Sequence Read Archive (SRA) database at NCBI with the accession number PRJNA521094. Supplemental material available at figshare: https://doi.org/10.25387/g3.10287660.

## Results

### Histological structure of feather follicles at different stages

**Figure 1:**
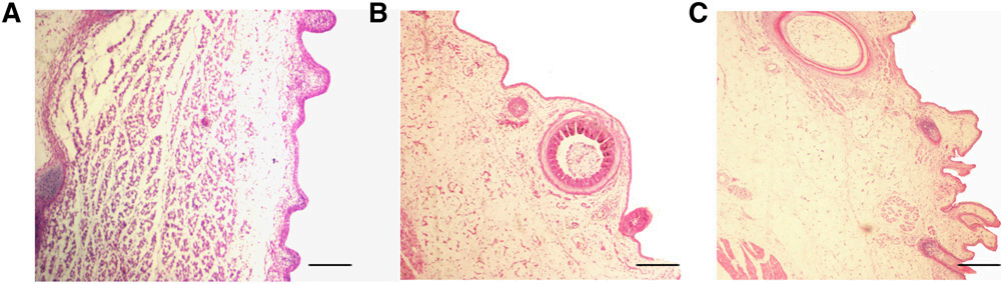
Microscopic observation of goose embryo skin at three different developmental stages. (A) primordial period of primary feather follicles at embryonic day 14; (B) primordial period of secondary feather follicles at embryonic day 18; (C) greater developmental period of secondary feather follicles at embryonic day 28. Magnified: 100×; Bar: 100 μm.

The histological sections examining skin morphogenesis at three embryonic developmental stages (E14, E18 and E28) are shown in Figure 1. The results indicate that at the early embryonic stage of E14, short feather buds were clearly distinguishable in the feather tracts, which had formed by cell proliferation at the epithelium (Figure 1A). In addition, the skin transforms from a bud to a feather follicular structure and the period at E14 was categorized as the stage of primordial period of primary feather follicles. By E18 (primordial period of secondary feather follicles), the microstructures of feather follicles in the geese embryos showed that the development of primary and secondary follicles was independent, and there was no common organizational structure between them (Figure 1B). At E28 (greater developmental period of secondary feather follicles), the follicles were shaped into a deep pit with further invagination and the feather germs with the continued distal growth resembled a long cylinder sticking out of the follicles, and at this period, the muscles, nerves, glands and blood vessels connected with mature feather follicles gradually become abundant in the dermis (Figure 1C).

### Illumina sequencing, de novo assembly and functional annotation of the transcriptome

To obtain the transcriptome reference and confirm the DEGs at the three development stages (E14, E18 and E28), nine RNA-seq libraries (E14, E18, and E28 stages with three biological replicates, respectively) were constructed. Sequencing of the skin transcriptome resulted in a total of 445,962,272 raw reads by Illumina paired-end sequencing technology. After filtering out the low quality reads, we obtained 436,730,608 clean reads for further analysis, encompassing 643,532,597,01 total nucleotides (nt) (Table 1). Sequences gained from the Trinity were called unigenes, which are unique and have the capacity to form the clusters in which the similarity among sequences is >70%. A total of 56,301 unigenes were obtained by the *de novo* assembly, with the average length of 1,155 nt and N50 length of 2,743 nt.

**Table 1:** Characteristics of reads around filter for the nine transcriptome data

Sample	Before Filter Reads Num	After Filter Reads Num (%)	After Filter Data (nt)	Reads Len	GC	Adapter (%)	low quality (%)
E14-1	49939458	48876816 (97.87%)	7204083186	150	56.92%	221238 (0.44%)	836566 (1.68%)
E14-2	51519416	50431878 (97.89%)	7433794979	150	56.75%	234176 (0.45%)	848442 (1.65%)
E14-3	56196964	55109180 (98.06%)	8126867450	150	57.19%	240648 (0.43%)	841756 (1.5%)
E18-1	44984808	44046244 (97.91%)	6494594765	150	56.91%	215968 (0.48%)	718410 (1.6%)
E18-2	47474146	46525366 (98%)	6864444469	150	56.92%	199728 (0.42%)	744528 (1.57%)
E18-3	48093160	47145862 (98.03%)	6954709712	150	56.70%	207754 (0.43%)	735074 (1.53%)
E28-1	50660254	49628426 (97.96%)	7306941403	150	56.82%	234454 (0.46%)	792682 (1.56%)
E28-2	47186850	46097640 (97.69%)	6772563222	150	59.08%	224288 (0.48%)	860350 (1.82%)
E28-3	49907216	48869196 (97.92%)	7195260515	150	57.78%	219620 (0.44%)	813654 (1.63%)
In Total	445962272	436730608 (97.93%)	64353259701				

**Figure 2:**
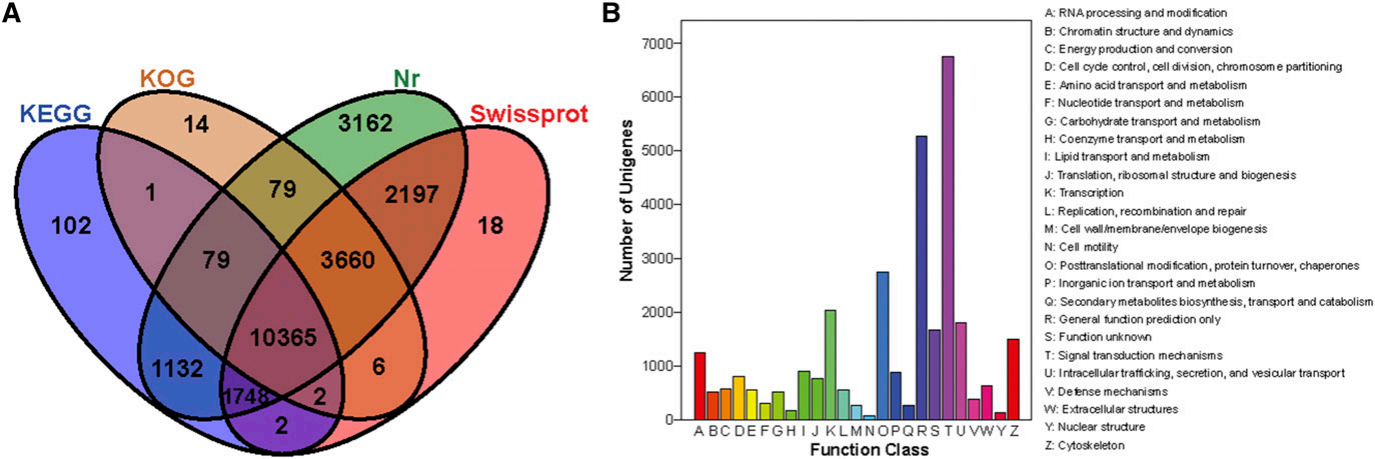
The distribution and KOG classification of annotated unigenes of the *Anser cygnoides*. (A) Venn diagram showing the number of unigenes annotated to the four databases (Nr, Swissprot, KOG and KEGG). (B) Clusters of KOG function classification of the *de novo* assembled unigenes. 14,206 unigenes were annotated and assigned to 25 function categories.

The acquired unigenes were annotated by BLAST searches against NCBI Nr database, Swiss-Prot database, KEGG database and KOG database using a cut-off E-value of 10-5 (Figure 2A). 17,998 (31.97%) and 13,431 (23.86%) unigenes were annotated using the Swiss-Prot database and KEGG database, respectively. 56,301 unigenes were subjected to a search against the Nr database, among them, 22,422 (39.83%) unigenes were noted on the Nr database, through which we could obtain the similarity of function information and gene sequence between *Anser cygnoides* and other species. The E-value distribution and the top 10 species distribution are shown in Table S2. KOG is a database where we could classify the orthologous gene products and evaluate the possible functions of unigenes. Based on the orthologous relationship, 14206 (25.23%) unigenes were aligned to the KOG database and classified into 25 KOG categories (Figure 2B, Table S3). Out of the 25 KOG groups, the largest group was “Signal transduction mechanisms” with 6752 unigenes, followed by “General function prediction only” (5271 unigenes), “Posttranslational modification, protein turnover, chaperones” (2745 unigenes), “Transcription” (2039 unigenes), and “Intracellular trafficking, secretion, and vesicular transport” (1806 unigenes). Only 67 unigenes were enriched to the “Cell motility”. The same ortholog gene function in the KOG classification might help us analyze the functions of DEGs related to the development of feather follicles.

### Identification of DEGs

**Figure 3:**
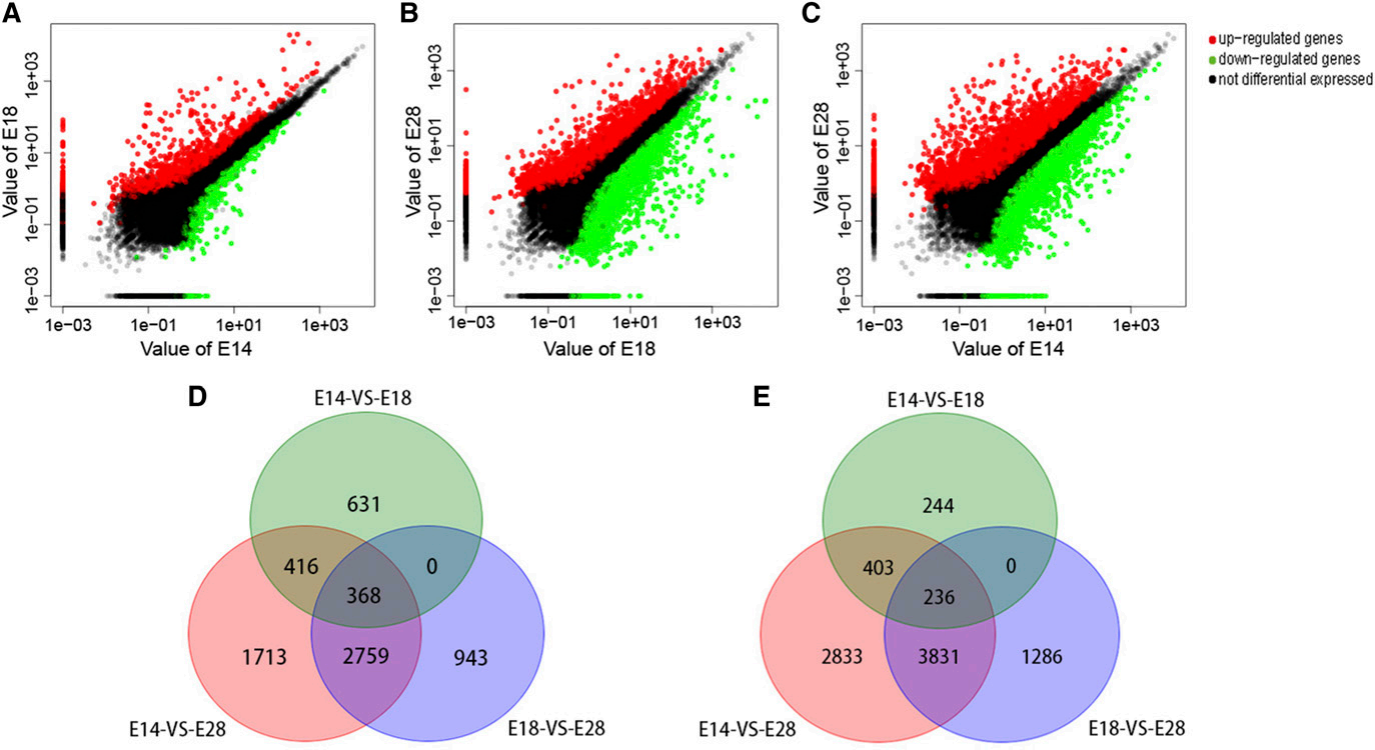
Comparison of the DEGs in three feather follicles developmental stages. A-C: scatter plot of the DEGs in three combinations. The x-axis and y-axis represent the DEGs expression levels of the two different development stages, respectively. (A) E14 *vs.* E18; (B) E18 *vs.* E28; (C) E14 *vs.* E28. (D) Venn diagram of the up-regulated DEGs. (E) Venn diagram of the down-regulated DEGs. The figures in the circle indicate the number of unique or shared DEGs among the comparisons of E14 *vs.* E18, E18 *vs.* E28 and E14 *vs.* E28.

Digital gene expression tags were used to identify the DEGs. As the feather follicles develop from E14 to E18, 2,298 DEGs are differentially expressed, of which 1,415 are up-regulated and 883 are down-regulated (Figure 3A). Similarly, E28 with visible alteration of the secondary feather follicles development in comparison to E18 is characterized by 9,423 DEGs of which 4,070 are up-regulated and 5,353 are down-regulated (Figure 3B), while 5,256 DEGs identified from E14 to E28 were up-regulated and 7,303 down-regulated (Figure 3C). However, a number of DEGs were stage-specific, which were only specifically expressed between two developmental stages but not between the other consecutive stages. To obtain these DEGs, we did up/down-regulated Venn diagrams. Among them, there were 631, 943, and 1713 stage-specific DEGs with up regulation in E14 *vs.* E18, E18 *vs.* E28, and E14 *vs.* E28, respectively (Figure 3D), and there were 244, 1286, and 2833 stage-specific DEGs with down regulation in E14 *vs.* E18, E18 *vs.* E28, and E14 *vs.* E28, respectively (Figure 3E). The identified DEGs with stage-specific up expression in E14 *vs.* E18, E18 *vs.* E28, and E14 *vs.* E28 were listed in Table S4 and the DEGs with stage-specific down expression were listed in Table S5.

### Gene ontology (GO) analysis of skin transcriptome

**Figure 4:**
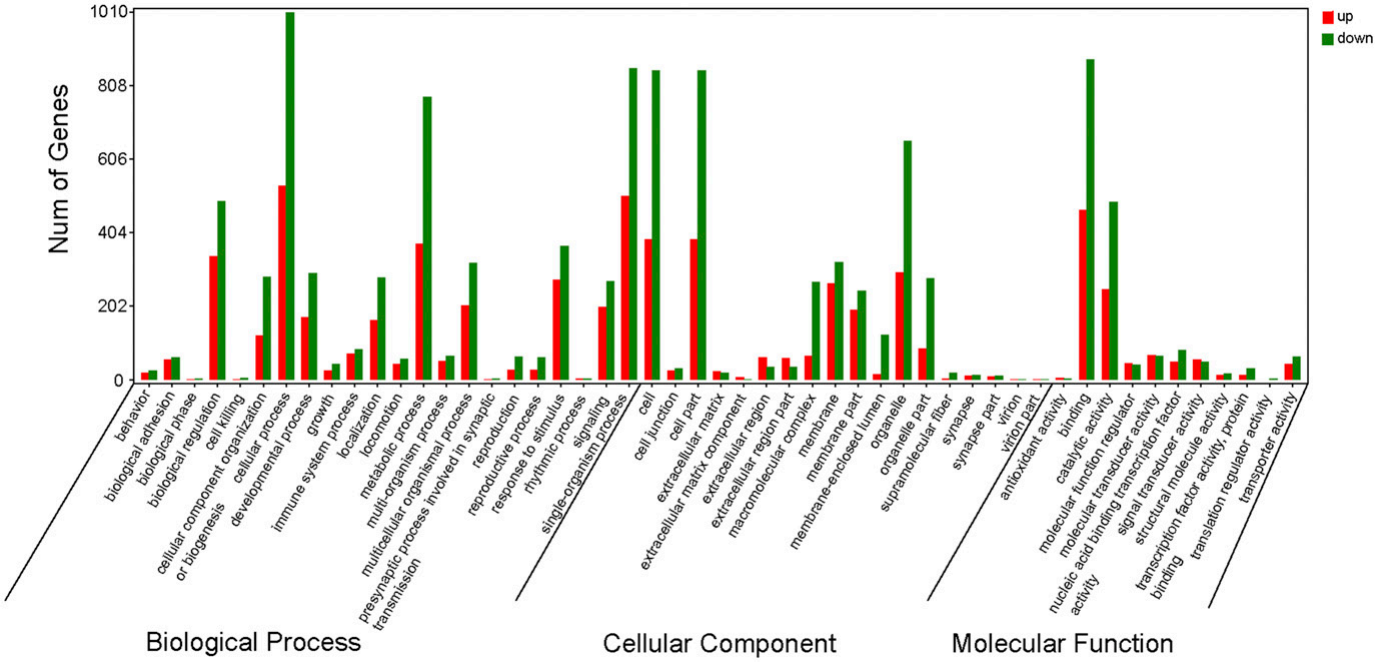
Distribution of the GO term categories for differentially expressed unigenes in E18 *vs.* E28. The GO annotation terms are divided into three main categories: biological processes, cellular components and molecular functions. The ordinate represents the number of genes classified into the corresponding function term.

Gene functions can be described by GO analysis results. Hence, we determined the significant enriched GO terms (*P* < 0.05) related to the feather follicles development from the DEGs in E14 *vs.* E18, E18 *vs.* E28, and E14 *vs.* E28. According to the GO annotation, the DEGs were divided into three major GO categories: cellular component, biological process, and molecular function. In E14 *vs.* E18, 1,162 DEGs were categorized into different functional groups, among which 351 DEGs were assigned to cellular component, 424 DEGs were assigned to biological process, and 387 DEGs were assigned to molecular functions (Table S6). In E14 *vs.* E28, 6,780 DEGs classified into three functional categories were assigned to cellular component (2,067 DEGs), biological process (2,493 DEGs), and molecular function (2,220 DEGs) (Table S7). In E18 *vs.* E28, a total of 5,226 DEGs were categorized into different functional groups. The cellular component category (1,578 DEGs), had a high percentage of genes from categories of “cell”, “cell part”, and “organelle”. In the biological process category (1,929 DEGs), “cellular process”, “single-organism process”, and “metabolic process” were the prevailing terms. In the molecular function category (1,719 DEGs), the dominant subcategories were those genes associated with “binding”, “catalytic activity”, and “molecular transducer activity” (Figure 4, Table S8).

### Series-cluster and KEGG pathway enrichment analysis

**Figure 5:**
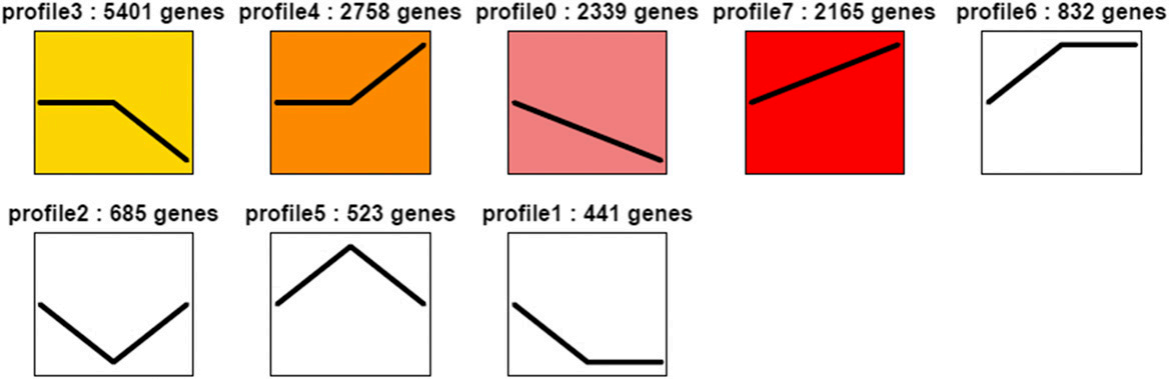
DEGs expression profiles across the three development stages. The profiles with color (*P* < 0.05) mean significant enrichment, conversely, non-significant enrichment. Profiles with the same expression tendency are similar in color.

The diverse and complex interactions of genes can be indicated by the expression profiles. Genes with similar functions may belong to the similar expression profiles. To better characterize the significant expression profiles associated with the feather follicles development, 15,144 DEGs using the same analysis were clustered into eight profiles with distinctive expression patterns (Figure 5). And all the profiles were showed according to the number of DEGs. Profile 3 contained the largest number of DEGs (5401, 35.66%) maintaining a relatively stable expression from E14 to E18, and then decreased at E28. DEGs in E18 *vs.* E28 may play a central regulatory role in the quality and production of the down feathers. However, there is no significant difference in DEGs from E14 to E28 that was enriched in profiles 1, 2, 5, and 6, so profiles 0, 3, 4, and 7 were chosen for subsequent analysis.

The KEGG annotation and the cluster analysis of profiles help us understand the pathway enrichment analysis. According to the KEGG database, 2,424 DEGs were assigned to 198 KEGG pathways. The top 15 pathways with the highest representation of the DEGs associated with feather follicles development are shown in Table 2. The most abundant pathways were metabolic pathways (562, 23.18%), followed by biosynthesis of secondary metabolites (174, 7.18%) and focal adhesion (157, 6.48%). In addition, MAPK (mitogen-activated protein kinase) signaling pathway (ko04010), endocytosis (ko04144), cytokine-cytokine receptor interaction (ko04060), Wnt signaling pathway (ko04310), and cell adhesion molecules (CAMs) (ko04514) were also significantly enriched. The 45 of 892 unigenes (5.04%) in profile 3 and the 18 of 414 unigenes (4.35%) in profile 0 were annotated to Wnt signaling pathway.

**Table 2:** KEGG pathway enrichment analysis associated with feather development within 4 significant enrichment clusters

Pathway	ID	All_Profiles (2424)	Profile 0 (414)	Profile 3 (892)	Profile 4 (472)	Profile 7 (368)
Metabolic pathways	Ko 01100	562 (23.18%)	74 (17.87%)	217 (24.33%)	118 (25.00%)	73 (19.84%)
Biosynthesis of secondary metabolites	Ko 01110	174 (7.18%)	25 (6.04%)	54 (6.05%)	39 (8.26%)	28 (7.61%)
Focal adhesion	Ko 04510	157 (6.48%)	12 (2.90%)	30 (3.36%)	47 (9.96%)	45 (12.23%)
Neuroactive ligand-receptor interaction	Ko 04080	134 (5.53%)	16 (3.86%)	38 (4.26%)	31 (6.57%)	29 (7.88%)
MAPK signaling pathway	Ko 04010	117 (4.83%)	19 (4.59%)	30 (3.36%)	31 (6.57%)	19 (5.16%)
Biosynthesis of antibiotics	Ko 01130	110 (4.54%)	20 (4.83%)	40 (4.48%)	24 (5.08%)	16 (4.35%)
Endocytosis	Ko 04144	106 (4.37%)	11 (2.66%)	42 (4.71%)	21 (4.45%)	22 (5.98%)
Regulation of actin cytoskeleton	Ko 04810	105 (4.33%)	18 (4.35%)	22 (2.47%)	29 (6.14%)	19 (5.16%)
ECM-receptor interaction	Ko 04512	100 (4.13%)	8 (1.93%)	18 (2.02%)	30 (6.36%)	33 (8.97%)
Purine metabolism	Ko 00230	97 (4.00%)	20 (4.83%)	33 (3.70%)	16 (3.39%)	17 (4.62%)
Calcium signaling pathway	Ko 04020	90 (3.71%)	15 (3.62%)	21 (2.35%)	18 (3.81%)	25 (6.79%)
Adrenergic signaling in cardiomy-ocytes	Ko 04261	90 (3.71%)	10 (2.42%)	11 (1.23%)	26 (5.51%)	25 (6.79%)
Cytokine-cytokine receptor interaction	Ko 04060	90 (3.71%)	11 (2.66%)	19 (2.13%)	29 (6.14%)	21 (5.71%)
Wnt signaling pathway	Ko 04310	86 (3.55%)	18 (4.35%)	45 (5.04%)	7 (1.48%)	11 (2.99%)
Cell adhesion molecules (CAMs)	Ko 04514	82 (3.38%)	16 (3.86%)	20 (2.24%)	27 (5.72%)	16 (4.35%)

### DEGs with GO enrichment analysis in the pathways associated with feather follicles development

**Figure 6:**
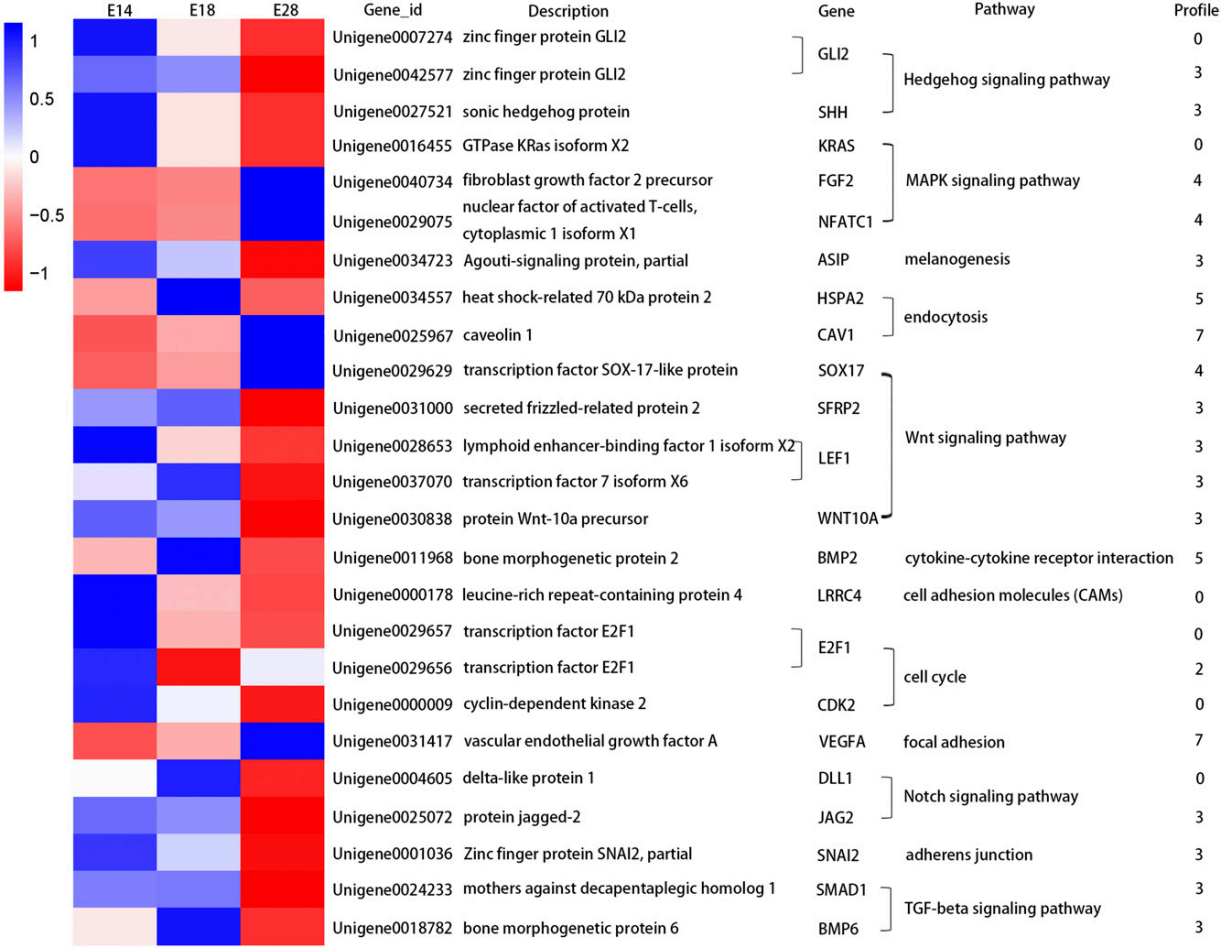
Heat map diagram of DEGs annotated in pathways related to feather development. Data on gene expression levels were standardized as z-score.

To focus on analysis of DEGs related to skin and feather follicles development, eight relevant specific GO terms such as epithelium development, regulation of keratinocyte proliferation, epithelial cell proliferation and so on were enriched in profiles 0, 3, 4, and 7 (Table 3). Besides, a total of 25 unigenes were annotated in the, Hedgehog signaling pathway MAPK signaling pathway, melanogenesis, endocytosis, Wnt signaling pathway, cytokine-cytokine receptor interaction, cell adhesion molecules (CAMs), cell cycle, focal adhesion, Notch signaling pathway, adherens junction, and TGF (transforming growth factor)-beta signaling pathway (Figure 6).

**Table 3:** DEGs annotated to different GO functions belonging to different profiles are involved in the development of feather follicles

Symbol	All profiles	Profile 0	Profile 3	Profile 4	Profile 7	GO function
*GLI2*	2	1	1	0	0	GO:0002009//morphogenesis of an epithelium
*SHH*	1	0	1	0	0	GO:0001942//hair follicle development
*FGF2*	1	0	0	1	0	GO:0060429//epithelium development
*CAV1*	1	0	0	0	1	GO:0048513//animal organ development
*SFRP2*	1	0	1	0	0	GO:0002009//morphogenesis of an epithelium
*WNT10A*	1	0	1	0	0	GO:0043588//skin development
*E2F1*	1	1	0	0	0	GO:0048513//animal organ development
*VEGFA*	1	0	0	0	1	GO:0050673//epithelial cell proliferation
*DLL1*	1	1	0	0	0	GO:0060429//epithelium development
*JAG2*	1	0	1	0	0	GO:0050789//regulation of biological process
*SNAI2*	1	0	1	0	0	GO:0010837//regulation of keratinocyte proliferation
*SMAD1*	1	0	1	0	0	GO:0048513//animal organ development
*BMP6*	1	0	1	0	0	GO:0060429//epithelium development

In the Wnt signaling pathway, 4 out of the 5 DEGs were clustered to profile 3 showing down-regulated trends between E18 and E28. They encode secreted frizzled related protein 2 (SFRP2, Unigene0031000), lymphoid enhancer binding factor 1 (LEF1, Unigene0028653 and Unigene0037070), and Wnt family member 10A (WNT10A, Unigene0030838). In contrast, only one DEG belonging to profile 4 was annotated as SRY-box 17 (SOX17, Unigene0029629) with the up-regulated trend between E18 and E28. In the MAPK signaling pathway, two DEGs were annotated as fibroblast growth factor 2 (FGF2, Unigene0040734) and nuclear factor of activated T cells 1 (NFATC1, Unigene0029075), belonging to profile 4. KRAS proto-oncogene, GTPase (KRAS, Unigene0016455), which encodes a protein that is a member of the small GTPase superfamily, was clustered to profile 0 showing the down-regulated expression pattern. In the Hedgehog signaling pathway, two DEGs annotated as GLI family zinc finger 2 (GLI2) were assigned to profile 0 (Unigene0007274) and profile 3 (Unigene0042577), and were both down-regulated from E18 to E28. Sonic hedgehog protein (SHH, Unigene0027521), associated with this pathway, was classified as belonging to profile 3 and also declined during the stage of secondary feather follicles development. Three DEGs were associated with the cell cycle pathway, two of which encoded E2F transcription factor 1 (E2F1, Unigene0029657 and Unigene0029656) were found to be differentially expressed belonging to profiles 0 and 2. In addition, the other one encoded cyclin-dependent kinase 2 (CDK2, Unigene0000009) and belonged to profile 0 which was identified as a down-regulated profile, suggesting that this gene might inhibit the development of the feather follicles in goose (Figure 6). In addition, combining the results of GO and KEGG analysis, DEGs probably related to the skin and feather follicles development in *Anser cygnoides* were identified and compared with our previous studies in *Anser anser* (Liu *et al.* 2018) by the values of RPKM and Count to show the expression of DEGs between the different species (Table S9).

### Validation of DEGs expression by RT-qPCR

**Figure 7:**
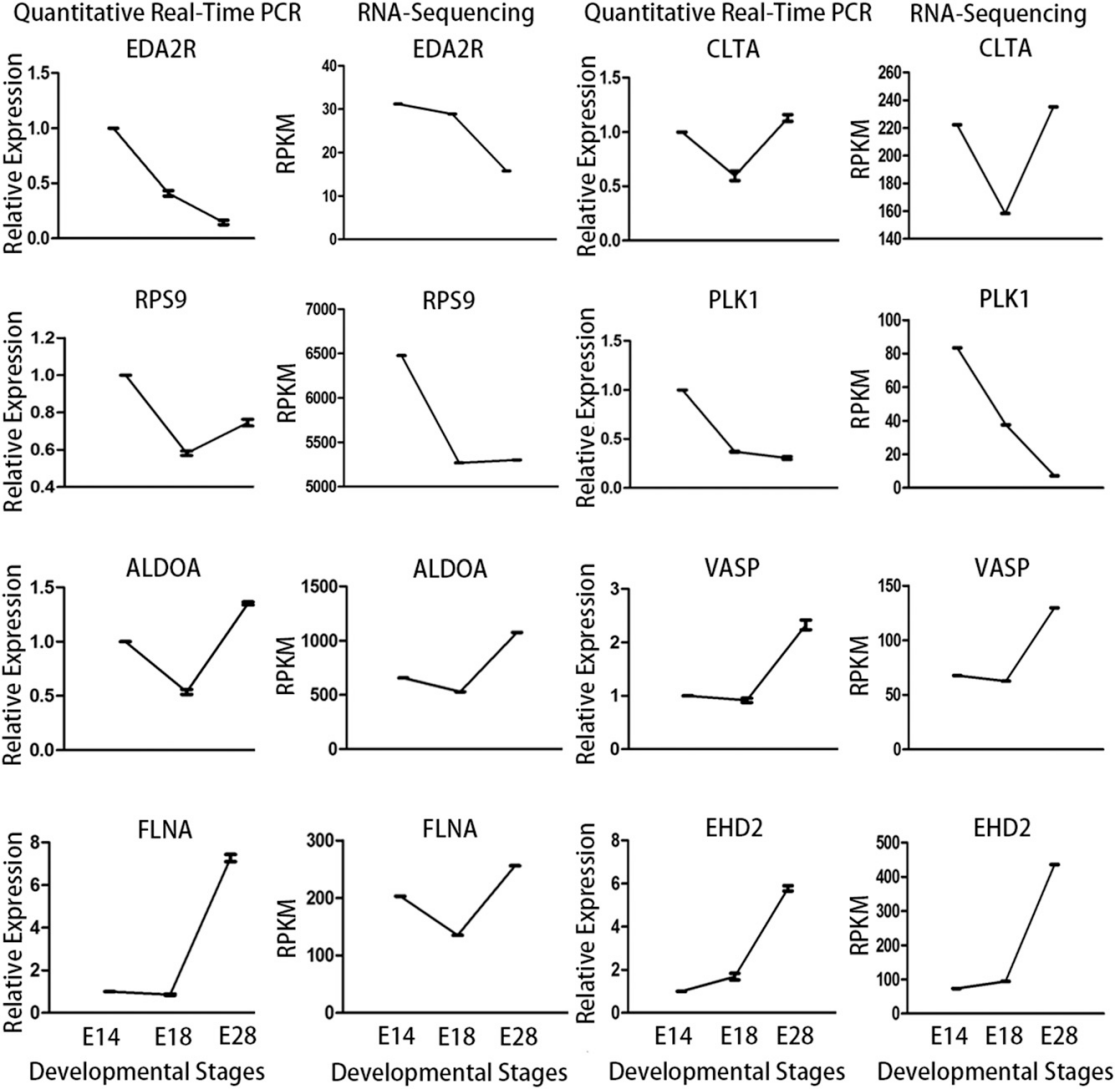
Validation of the RNA-Seq results by RT-qPCR through 8 selected DEGs. The first and third column: RT-qPCR results; the second and forth column: RNA-Seq results. The RT-qPCR data are represented as means ± SE of three replicates.

We used RT-qPCR to validate the expression of the selected DEGs during the three stages of feather follicles development. As shown in Figure 7, the patterns of expression of the DEGs identified using RT-qPCR are similar to those obtained from RNA-Seq indicating that RNA-Seq method used to quantify the expression profiles was reliable and accurate.

## Discussion

In the embryo, one of the crucial bioprocesses is the feather follicles development especially the secondary feather follicles which determines the commercial value of the down feathers. Lu *et al.* (2015) acquired the whole goose-genome draft sequence by shotgun sequencing and revealed the susceptibility to fatty liver according to the transcriptome analysis. In this study, we conducted *de novo* transcriptome analysis to investigate the complex molecular mechanisms underlying the skin and the feather follicles development in *Anser cygnoides* geese species. The transcript profiles were characterized at three distinct developmental stages (E14, E18 and E28) to identify the unigenes annotated against the known sequences and to lay the foundation for identifying candidate genes which are potentially involved in the feather follicles development. After assembly, the average lengths of the unigenes (1155 bp) were significantly longer than those of Taiwan County Chicken (ranging from 300 to 400 bp) (Ng *et al.* 2015), Liaoning cashmere goat (416 bp) (Liu *et al.* 2013), and Chinese Sika deer (136 bp) (Yao *et al.* 2012). These differences suggest that longer unigenes without gaps in the sequences were obtained and the transcriptome provided abundant unigene information to enrich the available genomic resources for *Anser cygnoides*.

For normal development of intrafollicular epidermis, hair follicles, other epidermal lineages and the space-time coordination of different signaling pathways are applied in complex processes of skin and feather morphogenesis (Amberg *et al.* 2019). KEGG, as an integrated database can be used for comprehensive analysis and biological interpretation of the gene functions (Han *et al.* 2013). As a large modular network, MAPK pathway regulates multiple physiological processes including the cell growth and differentiation (Junttila *et al.* 2008). And the interactions of MAPK signaling play a crucial role in the keratinocyte differentiation, which can also adjust the skin homeostasis to maintain the various functions of the epidermis (Schmidt 2001). Previous studies had indicated that MAPK signaling pathway is widely required in various aspect of hair, feather, and wool follicles cycle and morphogenesis in cashmere goat (Yuan *et al.* 2013), Pekin duck (Chen *et al.* 2017), Tibetan sheep (Liu *et al.* 2013), and mice (Akilli Öztürk *et al.* 2015). In skin tissue formation, ECM (extra-cellular matrix) signaling can regulate the properties and orchestrate functions of epithelial cells (Teräväinen *et al.* 2013). ECM receptor interaction regulates many physiological activities of cells such as the proliferation and migration, which is also deemed to associate with the initiation and the morphogenesis of skin appendages such as hair and feathers (Ariza de Schellenberger *et al.* 2011; Kaplan and Holbrook 1994). Special and highly developed dermal ECM commonly underlines epithelial cells whose dynamic regulations are vital for skin functions and development (Nyström *et al.* 2018). Wingless-types (Wnts), a large family composed of secreted glycoproteins, regulate the developmental and post-developmental physiology through various cellular processes (Golestaneh *et al.* 2009). In all parts of hair follicle morphogenesis, Wnt signaling is regarded as a significant signaling pathway for initiating hair bud formation in hair follicle stem cells proliferation and differentiation (Kwack *et al.* 2011). During the embryogenesis process, Wnt signaling protein family is related to a variety of developmental events, together with maintaining the induction capacity of the hair follicle, especially in establishing hair follicles and activating bulge stem cells to promote hair growth (Logan and Nusse 2004; Lin *et al.* 2014). In this study, different KEGG pathways with DEGs were identified including, Wnt signaling pathway (Ko04310, 86 DEGs), ECM-receptor interaction (Ko04512, 100 DEGs), and MAPK signaling pathway (Ko04010, 117 DEGs) which were predominant. These findings suggest that there may be an effective interaction among these pathways which may take part in the proliferation, differentiation, and apoptosis of the cells related to skin development and influence the formation and development of the feather follicles in *Anser cygnoides*.

The formation of hair or feather follicles is triggered by signals from the dermis that resulted in the formation of epidermal signaling center. Houghton *et al.* (2005) reported that the epidermal placode which are necessary to maintain and sustain the morphogenesis of the hair follicle prototype. In the three sampled developmental stages of embryonic skin feather follicles, different signaling pathways such as Notch signaling pathway and a large number of DEGs were likely associated with skin and feather growth, although these DEGs belonged to different profiles with different expression trends. Notch signaling pathway is composed of four receptors (Notch1–4) and five ligands (JAG1–2, DLL1, DLL3-4), which is the crucial evolutionarily conserved mediators for skin or hair development and participates in epidermal differentiation regulation and hair cycle maintenance (Pagie *et al.* 2018; Lin *et al.* 2011). In skin epidermis, basal expression of Notch ligands such as delta like canonical Notch ligand 1 (DLL1) has an important function in controlling the transition between basal and suprabasal cells (Fuchs 2008). Combined with KEGG enrichment and cluster analysis from Notch signaling pathway, we screened two key genes (*JAG2* and *DLL1*) related to skin and feather follicle development. However, Lowell *et al.* (2000) found that *DLL1* is highly expressed in stem cell clusters of the basal layer in human epidermis. The study by the authors Estrach *et al.* (2008) has demonstrated that the proliferation and differentiation in interfollicular epidermis can be controlled by *DLL1*. Putative Notch ligands *JAG2* and *DLL1* act synergistically to regulate the numbers of sensory hair cells that form in the organ of Corti (Kiernan *et al.* 2005). In this study, *DLL1* and *JAG2* were annotated to the GO database and were categorized into different functional parts related to the skin and feather follicles development (*DLL1*, GO:0060429//epithelium development; *JAG2*, GO:0050789//regulation of biological process). As the stage-specific gene, *DLL1* had decreased expression during the three development stages (profile 0) and was only specifically expressed in E14 *vs.* E28, however, *JAG2* in profile 3 only decreased at the stage of secondary feather follicles development and specifically expressed down regulation between E14 *vs.* E28 and E18 *vs.* E28. *FGF2*, which can be detected in the epidermal placode of genetically normal embryonic feather buds, induces feathers in scaleless mutant skins (Song *et al.* 1996). In addition, the studies on dermal papilla cells cultured showed that *FGF2* treatment of dermal papilla cells enhanced the ability of hair follicle induction (Osada *et al.* 2007). Kawano *et al.* (2005) quantified the *FGF2* mRNA expression in adult mouse skin at various stages of the hair growth cycle and found the highest mRNA expression was at the 0 day after depilation. Some transcriptional regulator genes for feather follicle protein synthesis were remarkably up-regulated at E18 *vs.* E28 (profile 4) and were specifically expressed up regulation between E14 *vs.* E28 and E18 *vs.* E28 such as *FGF2* belonging to MAPK signaling pathway. The dynamic expression pattern of *FGF2* in feather follicles during different development stages motivated us to pose a hypothesis: that *FGF2* might play a regulatory role in various phases of secondary feather follicles development. Snail family transcriptional repressor 2 (SNAI2), which is also named as SLUG, extensively described as the developmental transcription factor in the Snail family that is expressed in the unperturbed adult murine epidermis, regulating multiple gene targets (Newkirk *et al.* 2008). After knocking out the *SLUG* gene, the growth of the postnatal hair on the mice appeared significantly delayed, and in the embryonic and postnatal hair follicles, *SNAI2* had orchestrated temporal and spatial regulation of gene expression (Parent *et al.* 2010). Furthermore, the vital function of *SNAI2* was also reported in the epidermal progenitor to maintain epidermis (Mistry *et al.* 2015). In feather follicles growth, we investigated the potential regulatory effects of *SNAI2* on the dynamic expression profiles of the feather follicles development. As expected, *SNAI2* in profile 3 was annotated to the regulation of keratinocyte proliferation (GO:0010837) and involved in the adherens junction pathway (ko04520) by GO and KEGG analysis, respectively, which was only down-regulated at the beginning of the developmental stage of secondary feather follicles and was specific down regulation between E14 *vs.* E28 and E18 *vs.* E28. Bone morphogenetic proteins (BMPs) belonging to the TGF-beta superfamily are dominant for skin development by regulating the proliferation, differentiation, and apoptosis of the keratinocyte, and are expressed in both epithelium and dermis (Lewis *et al.* 2014). *BMP6* (bone morphogenetic protein 6), with high expression level in the early anagen of hair follicles, has already been reported to have an inhibitory effect on hair follicle regeneration and telogen-anagen transition (Clavel *et al.* 2012; Wu *et al.* 2019). According to McDonnell *et al.* (2001), keratinocytes that were treated with *BMP6* induced the differentiation markers at the early stage instead of the later stage. In the *BMP6* transgenic mice, the increased frequencies of epidermal apoptotic in comparison to the unchallenged skin showed that *BMP6* could induce the keratinocytes apoptosis (Wach *et al.* 2001). Importantly, goose (*Anser cygnoides*) results, were similar to the results of above studies, which reported that *BMP6* in profile 3 annotated into the epithelium development (GO: 0060429) was down-regulated at the beginning of the secondary feather follicles initiation and was only specifically expressed in E18 *vs.* E28. These existing findings suggest that *BMP6* may act as a disincentive in the secondary feather follicles morphogenesis of *Anser cygnoides* via the apoptosis of keratinocytes related to skin development. Polo-like kinase 1 (PLk1) as the highly conserved serine-threonine kinase has important functions in the cell mitosis and the genomic stability (Zhang *et al.* 2007). *PLK1* is regarded as the proto-oncogene and the attractive cancer target, which is overexpressed in various range of malignancies such as melanoma, prostate cancer and gastro-instestinal cancer (de Cárcer *et al.* 2018; Bucur *et al.* 2014). However, there is limited information on the functions of *PLK1* for skin and feather follicles development. In order to get more insights into what roles the *PLK1* plays on the feather follicles development in goose, we conducted the RNA-Seq combined with RT-qPCR, and found that *PLK1* as the down-regulated DEG was commonly expressed in E14 *vs.* E18, E14 *vs.* E28, and E18 *vs.* E28. And *PLK1* in profile 0 had the highest expression level in E14 and the lowest expression level in E28. Meanwhile, *PLK1* was annotated to the pathway of cell cycle (ko04110) and the GO term of negative regulation of cellular process (GO: 0048523). These findings suggest that *PLK1* may inhibit the proliferation and differentiation of the cells associated with skin and feather follicles growth.

## Conclusions

In this study, we divided the period of embryonic feather follicles development in *Anser cygnoides* into three stages: the primordial period of primary feather follicles (E14), the primordial period of secondary feather follicles (E18), and the greater developmental period of secondary feather follicles (E28). *De novo* transcriptome analysis was performed by functional annotation and cluster analysis to identify the pathways (MAPK signaling pathway, ECM-receptor interaction, Wnt signaling pathway) and DEGs with specific temporal expression patterns (*DLL1* in profile 0, *FGF2* in profile 4, *SNAI2* in profile 3, *BMP6* in profile 3, *PLK1* in profile 0) which might play a regulatory role in primary or secondary feather follicles development and provide crucial information on candidate genes for further analysis. Overall, our results present a comprehensive characterization of gene expression profiles during the skin and feather follicles development process, which also provides a valuable resource for future down feather studies in agricultural economic growth.
